# Seasonal Variations of Serum Zinc Concentration in Adult Population: Tehran Lipid and Glucose Study

**Published:** 2019-08

**Authors:** Sahar ASKARI, Golaleh ASGHARI, Hossein FARHADNEJAD, Arash GHANBARIAN, Parvin MIRMIRAN, Fereidoun AZIZI

**Affiliations:** 1. Endocrine Research Center, Research Institute for Endocrine Sciences, Shahid Beheshti University of Medical Sciences, Tehran, Iran; 2. Student Research Committee, Nutrition and Endocrine Research Center, Research Institute for Endocrine Sciences, Shahid Beheshti University of Medical Sciences, Tehran, Iran; 3. Nutrition and Endocrine Research Center, Research Institute for Endocrine Sciences, Shahid Beheshti University of Medical Sciences, Tehran, Iran; 4. Prevention of Metabolic Disorders Research Center, Research Institute for Endocrine Sciences, Shahid Beheshti University of Medical Sciences, Tehran, Iran; 5. Endocrine Research Center, Research Institute for Endocrine Sciences, Shahid Beheshti University of Medical Sciences, Tehran, Iran

**Keywords:** Adult, Serum zinc, Trace elements, Seasonal variation

## Abstract

**Background:**

Zinc, an essential trace element, plays a key role in many biological human body functions. Serum zinc concentration is the most widely used indicator of zinc status for general populations. Considering the limited data available on seasonal fluctuation of serum zinc concentration, we aimed at determining seasonal variations in serum zinc concentrations of Tehranian adults.

**Methods:**

The current study was conducted within the framework of the Tehran Lipid and Glucose Study, on 4698 subjects, aged ≥20 years. Serum zinc samples of subjects were obtained from all four seasons over three years (from 2009 to 2011); samples of similar seasons over three years were placed in one group and the geometric means of serum zinc concentration of four seasons were compared to determine possible seasonal variations.

**Results:**

Participants with mean age 46.3 yr and geometric mean of serum zinc concentration 116.3 μg/dl, were studied for almost three years through four seasons. Serum zinc concentrations in spring and summer were significantly higher than those in autumn and winter (112.2 and 114.4 vs. 106.7 and 104.8 μg/dl; *P*<0.001, respectively). Moreover, monthly serum zinc concentration of all subjects differed, with the lowest and highest levels found in October and August (98.5 vs. 122.7; *P*<0.001).

**Conclusion:**

This study demonstrates the difference in serum zinc concentration in Iranian adults of both genders in different months and seasons during the year.

## Introduction

Zinc is the twenty-third most abundant element in the earth’s crust and the second most abundantly distributed trace element after iron in the body (1). A very minute amount of zinc plays a key role in many biological functions such as catalyzing enzyme activity, contribution in protein structure, DNA synthesis, insulin synthesis and release, cardiovascular homeostasis, and regulation of gene expression (2, 3). Zinc deficiency is one of the major risk factors for occurrence of diseases worldwide and is associated with a 1.8 million mortality rate annually (4). Prevalence of zinc deficiency had been reported to range between 2.3% and 7.1% among young Iranian adults, aged 18–30 yr (3, 5).

Concentration of serum zinc is tightly regulated and is not considered to be a specific and sensitive biomarker of zinc status in individuals; however, plasma zinc concentration is the most widely used indicator of zinc status for general populations and is the only biochemical indicator of zinc status that has adequate reference data (3, 6). Assessment of serum zinc concentrations along with evaluating dietary zinc intakes, can also helpful in determining zinc deficiency (3, 7). Furthermore, this indicator can be an applicable tool in the evaluation of the beneficial or adverse effects of zinc supplementation in general populations (7).

Although, several factors including age, sex, diet, smoking habits, alcohol consumption, and body mass index as important determinants of serum values of nutrients such as serum zinc concentration (8, 9); however, to date, there are limited data available on the seasonal variations of serum zinc, and what are available come from investigations conducted on animal samples such as fish, sheep, and dog with controversial findings (10–12). Levels of plasma zinc are positively correlated to temperature in various seasons (12). However, there is no study on this topic in humans, and only in two studies, the effects of seasonal variation of hair zinc have been assessed (13, 14), with one reporting that hair zinc levels were higher in the season with shortest daylight hours (13); the other found no seasonal variation in hair zinc concentration (14). To best of our knowledge, no study has assessed serum zinc concentrations based on seasonal variations in Iranian populations.

We aimed to investigate the seasonal variations of serum zinc concentration in a sample of Tehranians, who participated in the Tehran Lipid and Glucose Study (TLGS).

## Materials and Methods

### Study population

The current study was conducted within the framework of the TLGS, an ongoing study aimed at preventing non-communicable diseases (NCD) and developing programs promoting healthy lifestyles and reducing NCD risk factors (15). Baseline data for the TLGS were collected from 15005 participants, aged ≥3 yr, residents under the coverage of three medical health centers in District No. 13 of Tehran, the capital city of Iran. Participants were followed up every 3 yr to update their data on demographics and lifestyle, biochemical, clinical, and dietary measurements; the baseline survey was a cross-sectional study conducted from 1999 to 2001, and surveys 2 (2002–2005), 3 (2006–2008), 4 (2009–2011), and 5 (2012–2015) were prospective follow-up surveys.

For the current study, in the fourth survey of the TLGS (2009–2011), from among 10887 TLGS participants aged ≥20 yr, 5637 were randomly selected for assessment of serum zinc concentration. Participants with conditions that affect serum zinc concentrations like smoking (>1 cigarette per day or using water pipe) (8), taking medications, pregnancy, hospitalization since the past 3 months and diabetes mellitus, underlying malignancy (e.g. cancer), or diarrhea, were excluded from the study (n=939). Some individuals fell into more than one exclusion category. Finally, after exclusion, 4698 individuals, including 2778 females and 1920 males, age range 20–94 yr, remained in the study for assessment of serum zinc concentrations.

The protocol of this study was approved by the Ethics Committee of the Research Institute for Endocrine Sciences (RIES), affiliated to the Shahid Beheshti University of Medical Sciences, Tehran, Iran. Written informed consent was obtained from all participants.

### Evaluation of Serum Zinc Concentration

The method used for evaluation of zinc concentrations has been described in detail previously (3). After 12–14 h overnight fasting, 10 milliliters of venous blood were collected and transferred to the TLGS research laboratory; sera were separated by 10 to 15 min centrifuging and kept at −20 °C for further evaluation. Serum zinc concentrations were determined by flame atomic absorption spectrometry (CTA-2000, Chem Tech, Analytical Co., Kempston, UK). Serum zinc samples of subjects were obtained in all four seasons over the three years (from 2009 to 2011) and samples of similar seasons (three years) were placed in one group. Geometric means of zinc concentration of four seasons were compared to determine possible seasonal variations.

### Statistical analysis

**Fig. 1:**
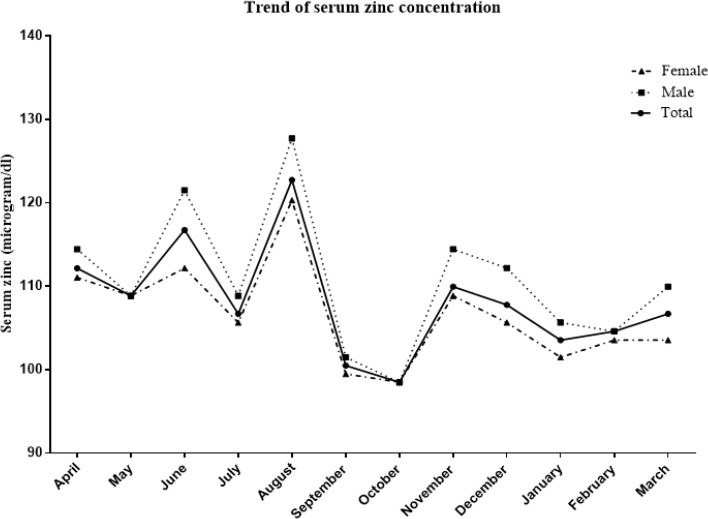
The trend of serum zinc concentration during the months of a year in all populations and either gender

SPSS (ver. 15.0; Inc., Chicago, IL, USA) was used for data analyses and *P*-values<0.05 were considered statistically significant. Normality of all variables distribution was evaluated by histogram charts and Kolmogorov-Smirnov analysis. Serum zinc concentrations of subjects were compared between seasons and months using ANOVA test and are expressed as the geometric mean ± standard error (SE), Post hoc Scheffe analysis test was used to assess the differences of serum zinc concentrations between seasons and months. The trend of serum zinc concentration for months of a year in all subjects and both genders is shown in Fig. 1.

## Results

Mean ± SD age of the study population (41% male) was 46.3 ±16.2 yr. Men were older than women in the study were population (47.3±16.8 vs. 45.6±15.6), a difference that was statistically significant (*P*=0.001). Zinc levels of males in the 20–29, 30–39, and 40–49 yr age groups were significantly higher than females in similar groups (*P*=0.001); however, in those aged ≥ 50 yr, serum zinc concentrations in women were higher than those of men. Table 1 presents the time trend of serum zinc concentrations for all samples and both males and females during four seasons of a year.

**Table 1:** Geometric means of serum zinc concentration (μg/dl) during the four seasons, based on gender

***Variable***	***Total***		***Male***			***Female***			
	***n***	***Geo mean***	***SE***	***N***	***Geo mean***	***SE***	***n***	***Geo mean***	***SE***
Spring	742	112.2^ab^	1.2	317	115.6^a^	1.9	425	109.9^ab^	1.7
Summer	1477	114.4^ab^	1.2	617	116.7^ab^	1.9	860	112.2^ab^	1.5
Autumn	1896	106.7	0.8	765	109.9	1.4	1131	104.6	1.1
Winter	1522	104.6	0.9	665	106.7	1.3	857	102.5	1.1
*P*-value			< 0.001			< 0.001	< 0.001		

Data are presented as mean (SE) and *P*-values are for the comparisons across groups, with the use of one way- ANOVA.

a Significantly different from Autumn, using post hoc Scheffe analysis test.

b Significantly different from Winter, using post hoc Scheffe analysis test.

Serum zinc concentration of all participants in spring and summer was significantly higher than during autumn and winter (112.2 and 114.4 vs. 106.7 and 104.6 μg/dl; *P*<0.001, respectively); seasonal differences were also observed between genders with the lowest and highest values being observed in men and women between winter and summer respectively (106.7 vs. 116.7 μg/dl in men and 102.5 vs. 112.2 μg/dl in women *P*<0.001). Monthly serum zinc concentrations significantly differed by sex groups (Table 2). The lowest value (98.5 μg/dl) in all participants, both men and women were for Oct. The highest serum zinc concentrations in all participants, both men and women were documented in Aug (122.7, 127.3, and 120.3; *P*< 0.001, respectively). Fig. 1 indicates the serum zinc trend in total population and both genders for all months of a year, revealing a peak in August for almost all populations and for both sexes (geometric mean of serum zinc was 122.7 for all participants, 127.3 for males and 120.3 μg/dl for females). Interestingly the lowest rate observed, directly after Aug (summer), in Oct (autumn), i.e. the geometric mean of serum zinc was 98.5 for all participants (98.5 for males and 98.5 μg/dl for females).

**Table 2:** Geometric means of serum zinc concentrations (μg/dl) based on sex during 12 months of the year

***Variable***	***Total subjects***		***Male***			***Female***			
	***n***	***Geo mean***	***SE***	***N***	***Geo mean***	***SE***	***N***	***Geo mean***	***SE***
April	198	112.2^ab^	2.6	77	114.4	3.9	121	111.0	3.6
May	264	108.8^c^	1.7	113	108.8^c^	2.5	151	108.8	2.4
June	280	116.7^dabefg^	2.3	127	121.5^abe^	3.5	153	112.1^ab^	2.9
July	414	106.7^c^	1.5	182	108.8^c^	2.2	232	105.6^c^	2.1
August	839	122.7^abhiefg^	1.7	346	127.7^abhiefg^	2.9	493	120.3 ^abief^	2.1
September	575	100.5^hi^	1.2	230	101.5^h^	2.0	345	99.5	1.6
October	224	98.5^hi^	3.3	89	98.5^h^	5.2	135	98.5	4.3
November	763	109.9^e^	1.6	287	114.4	2.8	476	108.8	2.1
December	558	107.8	1.5	248	112.2	2.4	310	105.6	1.9
January	799	103.5	1.2	371	105.6	1.8	428	101.5	1.5
February	353	104.6	1.6	146	104.6	2.6	207	103.5	2.0
March	370	106.7	2.0	148	109.9	2.9	222	103.6	2.6
*P*-value			< 0.001			< 0.001	< 0.001		

Data are presented as mean (SE) and *P* values are for the comparisons across groups, with the use of one way- ANOVA.

a Significantly different from September, using post hoc Scheffe analysis test

b Significantly different from October, using post hoc Scheffe analysis test

c Significantly different from August, using post hoc Scheffe analysis test//

d Significantly different from July, using post hoc Scheffe analysis test //

e Significantly different from January, using post hoc Scheffe analysis test //

f Significantly different from February, using post hoc Scheffe analysis test

g Significantly different from March, using post hoc Scheffe analysis test //

h Significantly different from November, using post hoc Scheffe analysis test //

i Significantly different from December, using post hoc Scheffe analysis test

## Discussion

This study assessed seasonal variations of serum zinc concentrations in a relatively large healthy sample of Iranian adults using standard methods. To control for the effect of season on serum zinc concentrations, we compared zinc concentrations during different months of the year. The present study indicates that serum zinc concentrations in the spring and summer seasons are higher than in autumn and winter. Moreover, the level of serum zinc in months with short-light days was lower than in other months in all populations and both genders.

Plasma zinc concentration as an important indicator of zinc status in the general population can be influenced by some factors such as sex, age, diet, smoking status, and alcohol consumption (8). Changes of serum zinc concentrations in various seasons as a major direct indicator of zinc status in populations can determine the number of dietary zinc requirements in populations during the year. In our study, zinc levels in men were significantly different to those of women in the same age range, a difference explained by lower lean body mass and difference in sex hormones, i.e., estrogen and progesterone in women, compared to men (6).

To the best of our knowledge, there is no study to date, assessing seasonal variations of serum zinc concentrations in humans. Previous studies have mostly evaluated seasonal variations of dietary zinc intakes (16–20). In China, the amounts of energy, protein, carbohydrate, and zinc intakes were evaluated and results showed that these amounts were adequate; however, no significant difference was seen in the southern and northern parts of China between different seasons, or the seasonal zinc intakes (16). In contrast, significant seasonal variations were found in nutrient intakes such as zinc in pregnant women and the significant effects of nutrient intake on neonatal assessments in different seasons, suggesting seasonal nutrient variation may affect fetal development at a cellular level (17). Considering serum zinc concentration as an indicator of dietary zinc in large populations (3, 21), our results confirmed results of previous studies, which showed significant differences in zinc intake across different seasons.

In our study, the lowest level of serum zinc was seen during rainy and shorter daylight seasons, including autumn and winter, which may be a result of inadequate zinc intakes and poor dietary habits (low consumption of some zinc-rich foods such as meat, nuts and chocolates) or unhealthy behaviors (22, 23). Overall, 199 cases and 106 matched controls were evaluated to determine the antioxidant imbalance, including zinc, vitamins E, C and carotenoids in a Cuban population and observed the lowest concentration of zinc at the end of the rainy season (Oct) in comparison to other seasons (18). In a study, 94 male workers were screened twice during winter and summer in one year and findings showed that from summer to winter the mean values of zinc increased from 11.6 to 12.3, a difference that was statistically significant (20). On the other hand, 80 children, aged 6–8 yr were concluded and because animal products, protein, fat, and vitamin C are rarely found in the food basket of children, the absorption of zinc might be low and variations in food pattern did not result in seasonal changes in energy and nutrient intakes such as zinc (19).

Moreover, seasonal variations of zinc in hair have been described in different studies, e. g. investigating 34 subjects, aged 18–54 yr, month-by-month, reported no seasonal variations in hair zinc concentrations (14). In another study, a marked seasonal variation in hair zinc concentrations was reported in Canadian and African children (13), indicating that the level of zinc was highest in the season with shortest daylight hours and no association was reported between changes in hair zinc with changes in socio-economic and demographic status, although the changes in level of hair zinc was associated with intakes of protein, zinc and dietary fiber.

Zinc deficiency may cause a wide variety of pathological disorders including atherosclerosis, insulin resistance, skin disorders, delayed wound healing, and increased incidence of bacterial infections (5, 24, 25). Considering that genetic disorders, poor intake, and increased recommendation have been considered as causes of zinc deficiency (26), the assessment of zinc status of individuals using proper indicator e.g. plasma zinc concentration is necessary for early detection of zinc deficiency and its prevention. However, serum zinc concentration mostly reflects recent intake (27); it is also a potentially valuable indicator of nutritional assessment to predict beneficial or adverse responses to zinc supplements (7). Our study is the first to investigate the seasonal variations of serum zinc concentration. In the current study, the lower serum zinc concentrations in cold and rainy seasons in comparison to spring and summer indicate an inconsistent dietary supply of zinc in individuals over the year.

The strengths of this study include a relatively large sample size used for determining the association between serum zinc concentrations and seasonal variations, a topic investigated for the first line in humans. A limitation of this study was that we could not investigate serum zinc concentrations season by season to evaluate the effect of seasonal variations for each subject. Moreover, the zinc from dietary supplements was not taken into account.

## Conclusion

Serum zinc concentrations in spring and summer seasons are higher than in autumn and winter. In addition, serum zinc concentrations of subjects in months with short-light days was lower than days of the year. These results could be used to design future studies and also as a tool to evaluate effect of dietary supplements during different seasons.

## Ethical considerations

Ethical issues (Including plagiarism, informed consent, misconduct, data fabrication and/or falsification, double publication and/or submission, redundancy, etc.) have been completely observed by the authors.
